# *Smoothened* gene alterations in keratocystic odontogenic tumors

**DOI:** 10.1186/1746-160X-10-36

**Published:** 2014-09-05

**Authors:** Zhang Rui, Peng Li-Ying, Qu Jia-Fei, Hong Ying-Ying, Chen Feng, Li Tie-Jun

**Affiliations:** 1 Department of Oral Pathology, Peking University School and Hospital of Stomatology, 22 Zhongguancun Avenue South, Haidian District, Beijing 100081, China; 2 Central Laboratory, Peking University School and Hospital of Stomatology, 22 Zhongguancun Avenue South, Haidian District, Beijing 100081, China

**Keywords:** Keratocystic odontogenic tumor (KCOT), Hedgehog (HH) signaling pathway, Gene mutation, *SMO* gene

## Abstract

**Background:**

It has been widely demonstrated that the hedgehog pathway is strongly associated with basal cell carcinoma of the skin (NBCCS). To assess potential DNA alterations related to keratocystic odontogenic tumors (KCOTs), we sequenced smoothened (SMO) genes in 12 sporadic KCOTs.

**Methods:**

Polymerase chain reaction (PCR), capillary electrophoresis and dideoxy chain-termination sequencing were used to examine potential DNA alterations in sporadic KCOTs.

**Results:**

Five alterations in SMO genes were detected. Four of these mutations consisted of two synonymous and three missense mutations; two of which have not been reported to date (c.T776A, c.T1281G).

**Conclusions:**

SMO genes may play an important role in the sonic hedgehog (SHH) pathway and could also be responsible for generating KCOTs and NBCCS. However, their influence on SHH signaling remains to be elucidated.

## Background

Odontogenic keratocyst (OKC) is an aggressive, cystic jaw lesion with strong growth potential and a high recurrence rate. In recent years, the World Health Organization (WHO) revised its name to keratocystic odontogenic tumor (KCOT). This reclassification is based on its aggressive behavior and high recurrence rate, emphasizing that KCOT is a benign tumor rather than a cyst [1-3]. Although the great majority of keratocysts occur in isolation as single, non-syndromic cysts, they may also present as multiple cysts as a feature of the nevoid basal cell carcinoma syndrome (Gorlin syndrome, OMIM#109400) [4].

At present, there are many manuscripts that focus on the relationship between KCOT and PTCH1 (patched) gene mutations, demonstrating that PTCH1, the gene responsible for NBCCS, may also play an important role in sporadic KCOTs [5-8]. The PTCH1 gene is a tumor suppressor gene located at 9q22.32 [9]. A study of 14 patients with NBCCS-associated KCOTs and 29 patients with sporadic KCOTs indicated that mutations in transmembrane 2 (TM2) are closely related to the development of sporadic KCOTs [5].

The hedgehog (HH) signaling pathway is a key regulator of embryonic development, controlling both cellular proliferation and cell fate. Binding of sonic hedgehog (SHH) to its receptor, patched (PTCH1), is believed to relieve normal inhibition by PTCH1 of smoothened (SMO), a seven-span transmembrane protein with homology to a G-protein-coupled receptor [10].

SMO is a tumor-related gene located at 7q32.3, contains 12 exons spanning approximately 24 kb, and encodes a 787-amino-acid transmembrane glycoprotein [11]. Its receptor is a G protein-coupled receptor that interacts with Patched, an important part of the HH signaling pathway during embryogenesis as well as adulthood [12,13]. The HH pathway has been demonstrated to play an important role in different development-related cancers [14-18], but the exact mechanism of action has not yet been elucidated. The protein generated by SMO is downstream of PTCH1; that is, the expression of PTCH1 restrains the activation of SMO, and thereby inhibits activation of the HH pathway [19-22]. Recent studies have highlighted the therapeutic value of SMO antagonists for the treatment of HH-linked cancers [22,23]. SMO, the main activator of the HH pathway may serve as a catalyst during the generation of cysts, and therefore, genetic mutations of SMO are of great importance.

## Methods

### Tumor samples and clinical background

Fourteen KCOT samples with a definite diagnosis were acquired from clinical sources at Peking University School of Stomatology, Oral and Maxillofacial Surgery Department. Diagnoses were based on WHO classification of tumors: pathology and genetics of tumors of the head and neck [24]. All samples were from Chinese patients (eight males and six females). Ages varied from 10 to 58 years, with an average of 29.2 years. Experimental protocols used in this study were reviewed and approved by the Ethics Committee of the Peking University Health Science Center (Peking, China). Informed consent was obtained from all subjects.

### DNA isolation and mutation analysis

Genomic DNAs from epithelial cells and interstitial cells of tumors were isolated using a QIAamp DNA Mini kit (Qiagen, Hilden, American) according to the manufacturer’s instructions. DNA was quantified using a Nanodrop (Thermo Fischer, Wilmington, DE). SMO (NM_005631.4) coding sequences were determined using polymerase chain reaction (PCR) and 1% agarose gel electrophoresis. Thirteen pairs of primers covering the entire coding sequences and a few nucleotides into the intron sequences on both ends of SMO are described in Table 1. PCRs were performed on a PTC-100 (MJ Research, Watertown, MA, USA) in a final volume of 30 μL containing approximately 100-ng template DNA, 200 μmol/L dNTPs, 0.2–0.4 μmol/L each primer, 1.25 U ExTaq polymerase (TaKaRa, Dalian, China), and 10X PCR buffer (50 mmol/L KCl, 10 mmol/L Tris–HCl, pH 9.0, and 1.5 mmol/L MgCl_2_). Exon 1, exon 12a and exon 12b of the SMO gene were amplified by a touchdown (TD) PCR technique (Table 1). For example, TD 65-56°C was performed as follows: an initial denaturation step at 95°C for 5 min, followed by 40 cycles at 94°C for 30 s, 30 s at an annealing temperature decreasing from 65°C to 56°C (decrease of 1°C each 2 cycles, 20 cycles at 55°C), 30 s at 72°C, and a final extension step at 72°C for 10 min. The specificity of amplified products was confirmed by 2% agarose gel electrophoresis. PCR products were purified by Life Technologies (Applied Biosystems, USA) and sequenced by an ABI 3730xl DNA sequencer (Applied Biosystems, USA). Both forward and reverse strands were sequenced. Any mutation detected was confirmed using both forward and reverse primers.

**Table 1:** **PCR conditions of *SMO *** **exons**

**Exon**	**Size(bp)**	**Forward primer sequence (5′to 3′)**	**Reverse primer sequence (5′to 3′)**	**Reference**	**Annealing temperature (°C)**
1	391	GGGCTGCTGCTGCTGCTG	GTCCCCGCCCTCTCCAAAACT	Sun et al., 2008 [25]	TD60 ~ 50
2	283	AAGAACTGTCCTGCCCAGATG	CCACTGGACCCTGCCCTATAC	Wang et al., 2013 [12]	63
3	305	AATAATTTGCCAAGCCAGCC	CTTCTGATCATGACCCTTCCC	Wang et al., 2013 [12]	63
4	256	AGGGTCATGATCAGAAGGGTC	AGTATGCAGTAGGGCAGAGCC	Wang et al., 2013 [12]	63
5	302	CTGACTTCTGGGAACCTCCAG	GACAGAAGGTGGGTTACTGGC	Wang et al., 2013 [12]	63
6	206	GTGGCGCAGGTATAGTGACTG	GCCCTATAGGAGCTAGCTGGG	Wang et al., 2013 [12]	63
7	175	GACTCCAGAGCCTTAGGACCC	TCCCTATGGCTAACTTGTCCC	Wang et al., 2013 [12]	63
8	196	AAGCAGTTCTTGGACTGAGCC	CCATCCATTGAATCTGCTGTC	Wang et al., 2013 [12]	55
9	379	AGTTGGAAGCTGCAGTGGG	CAAGGCTGTGCTAGAGGCAG	Wang et al., 2013 [12]	63
10	228	CTCTGGAAAGAATGGCATCG	TTCCAAATAATCTGTGTGCCC	Wang et al., 2013 [12]	55
11	215	AATGGCACTGACTATGGGAGG	CCACTCTTCAGATCCTCTGGG	Wang et al., 2013 [12]	63
12a	292	GAGCCAGGGCCCCAGGCTCGT	ACGCTCCCTGTCGGCAAGAGT	Sun et al., 2008 [25]	TD65-56
12b	315	AGTACCATTCCTCGACTGCCT	GGTATTGGTTCCTCTCTTTCC	Sun et al., 2008 [25]	TD65-56

### Statistical analysis

Experimental data were analyzed using the SPSS ver. 10.0 software and are presented as means ± standard deviation (SD) using statistical methods such as a *t*-test, analysis of bivariate correlation, *etc*. A P value <0.05 was considered to indicate statistical significance. Results are representative of two independent experiments.

## Results and discussion

### Hedgehog (HH) signaling pathway

Hedgehog (HH) is a signal transduction pathway closely related to cell growth and differentiation, and plays a vital role in embryonic development. Mutation or abnormal expression of components of this pathway will lead to various developmental defects and/or tumors. Altered HH signaling is implicated in the development of approximately 20-25% of all cancers, especially those of soft tissues [26]. These findings also suggest that proteins of the HH signaling pathway are predominantly located within the epithelial components of glandular odontogenic cysts (GOCs) and dentigerous cysts (DCs). Therefore, the HH signaling pathway may play an important role in the formation of epithelial lining [27]. The discovery of oncogenic mutations in the HH and mitogen-activated protein kinase (MAPK) pathways in over 80% of ameloblastomas, locally destructive odontogenic tumors of the jaw, was reported in 2014 by genomic analysis of archival material. Mutations in SMO are common in ameloblastomas of the maxilla, whereas BRAF mutations are predominant in tumors of the mandible [28]. In addition, SHH subgroup medulloblastomas are genetically distinct in infants, children and adults [29]. Most meningiomas have simple genomes, with fewer mutations, rearrangements and copy-number alterations than reported in other adult tumors. However, several meningiomas harbor more complex patterns of copy-number changes and rearrangements, including one tumor with chromothripsis. A subset of meningiomas lacking NF2 alterations harbored recurrent oncogenic mutations in AKT1 (p.Glu17Lys) and SMO (p.Trp535Leu), and exhibited immunohistochemical evidence of activation of these pathways [11]. In another study, SMO mutations, which activate the SHH signaling pathway, were identified in ~5% of non-NF2 mutant meningiomas. Collectively, these findings identify distinct meningioma subtypes [30]. Moreover, some findings provide a rationale to explore the use of SMO and BCL2 inhibitors as adjuvant therapy for treatment of DLBCL of the GC type [31]. The expression of SMO in NPC is generally high, whereas expression of PTCH-1 is relatively low. Downregulation of PTCH1 and upregulation of SMO may cause abnormal activation of the HH signaling pathway in NPC, albeit that the genesis and development of NPC may be associated with abnormal activation of HH signaling [32]. The HH signaling pathway is controlled by PTCH1 and SMO, which are located on the target cell’s membrane. As the receptor of sonic hedgehog (SHH), the PTCH1 gene (51 kb) encodes a 12-transmembrane-domain protein (total, 1,447 amino acids). There are two homologous genes in humans, PTCH2 and PTCH1, which negatively regulate SMO, a G-protein-coupled receptor, through SHH. SMO belongs to the G protein-coupled receptor FZ/SMO superfamily, containing a field that crosses the cell membrane seven times. Without a ligand (SHH), PTCH and SMO form an inhibitory compound, restraining signal transduction throughout the entire pathway. However, when SHH binds to PTCH1, SMO escapes from the inhibition by PTCH1, resulting in enhanced expression of downstream target genes, including the GLI superfamily [33]. As shown by numerous researchers, abnormal activation of the HH signaling pathway is closely related to the development of a variety of tumors, and both PTCH1 and SMO play a critical role in this pathway.

The HH signaling cascade is highly conserved and involved in the development of disease throughout evolution. Nevertheless, compared with other pathways, our mechanistic understanding of HH signal transduction is remarkably incomplete. In the absence of ligand, the HH receptor Patched (Ptc), represses the key signal transducer Smoothened (Smo) through an unknown mechanism. HH binding to Ptc alleviates this repression, causing Smo redistribution to the plasma membrane, phosphorylation and subsequent opening of the Smo cytoplasmic tail and Smo oligomerization. However, the order and interdependence of these events are poorly understood. We have mathematically modeled and simulated Smo activation for two alternative modes of activation, with Ptc primarily affecting either Smo localization or phosphorylation. Here, we show that Smo localization to the plasma membrane is sufficient for phosphorylation of the cytoplasmic tail in the presence of Ptc. Using fluorescence cross-correlation spectroscopy (FCCS), we also demonstrated that inactivation of Ptc by HH induces Smo clustering irrespective of Smo phosphorylation. Our observations therefore support a model of HH signal transduction whereby subcellular localization of Smo, and not phosphorylation, is the primary target of Ptc function [31].

Several studies [34-36] have confirmed that the PTCH1 gene is a tumor suppressor; this suggests that its mutation increases the likelihood of developing cancer, although few reports regarding the relationship between SMO and KCOT have been published.

### SMO mutations

Coding variants of SMO were identified following our analysis, and the mutations are listed in Table 2. The four SMO mutations detected were distributed on five exons (exons 2, 3, 5, 6, and 10), of which there were two synonymous mutations and three missense mutations.

**Table 2:** SMO mutations in 12 sporadic and 19 NBCCS-associated KCOTs

**Histology**	**Sample ID**	**Exon/intron**	**Nucleotide definition**	**Amino acid definition**	**dbSNP rs# cluster ID**	**MAF**	**Class**	**Function**
S*	K1, K2, K4,K5, K6, K7,K8, K10, K11	Exon 2	c.T776A	p.Phe166Ile	—	—	Missense	Unknown
S	K7	Exon 3	c.A862G	p. = (194)Glu	rs56334250	0.0266	Synonymous	Polymorphism
S	K4	Exon 5	c.T1281G	p.Val334Gly	—	—	Missense	Unknown
S	K2, K3, K4, K5, K6, K7, K8, K9, K10, K11, K12	Exon 6	c.G1444C	p. = (388)Gly	rs2228617	0.2277	Synonymous	Polymorphism
N*	N1, N2, N3, N4, N5, N6, N7, N8, N9, N10, N11, N12, N13, N14, N15, N16, N17, N18, N19,	Exon 6	c.G1444C	p. = (388)Gly	rs2228617	0.2277	Synonymous	Polymorphism
S	K1	Exon 10	c.G2049C	p.Ser590Thr	rs114406835	0.010	Missense	Polymorphism

*S = sporadic, N = NBCCS-associated.

**Figure 1:**
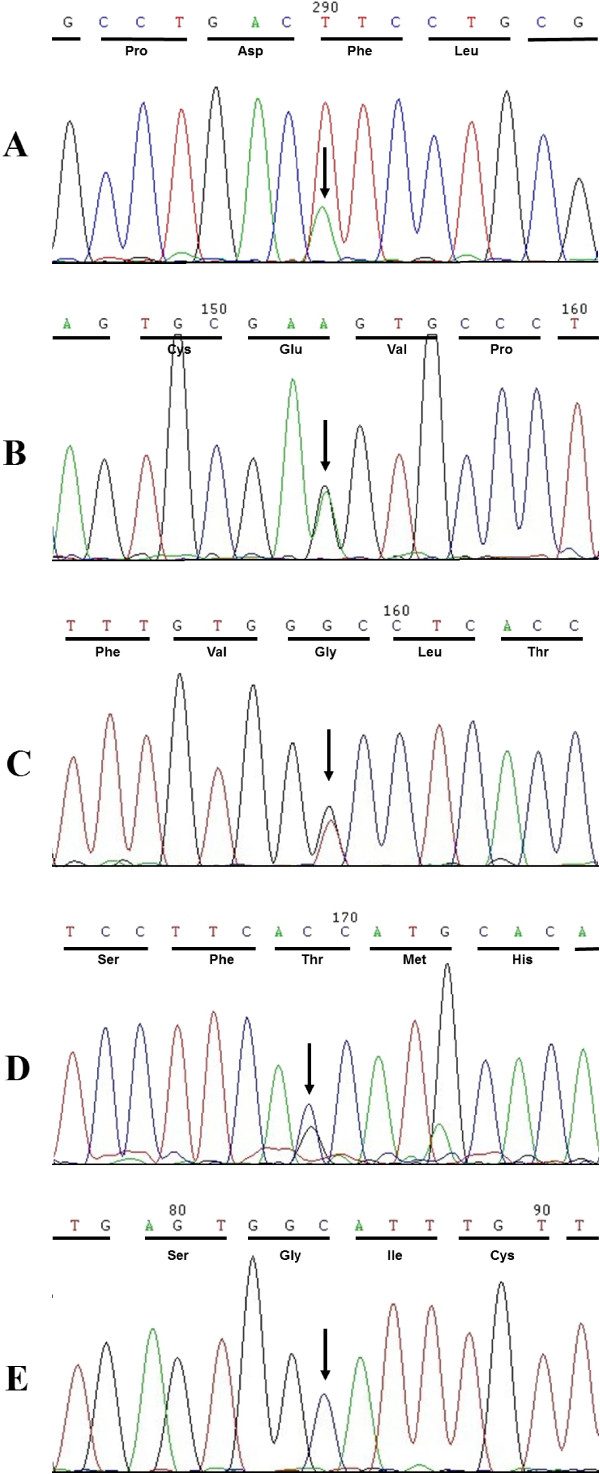
**SMO missense mutation in sporadic KCOTs. A**. This alternation at nucleotide position 776 of exon 2 is a heterozygous mutation from T to A and causes the p.Phe166Ile mutation. **B**. This alternation at nucleotide position 862 of exon 3 is a heterozygous mutation from A to G. However, this nucleotide change does not result in amino acid change (p. = (194) Glu). Minor allele frequency (MAF) = 0.0266. **C**. This alternation at nucleotide position 1444 of exon 6 was identified in seven tumors. Among 31 patients sequenced, 17 are homozygous and 13 are heterozygous at this site. This nucleotide change does not result in an amino acid change (p. = (388) Gly). MAF = 0.2277. **D**. This alternation at nucleotide position 2049 of exon 10 is a heterozygous mutation from G to C and causes the p.Ser590Thr mutation. **E**. This alternation at nucleotide position 1281 of exon 5 is a heterozygous mutation from T to C and causes the p.Val334Gly mutation.

**Figure 2:**
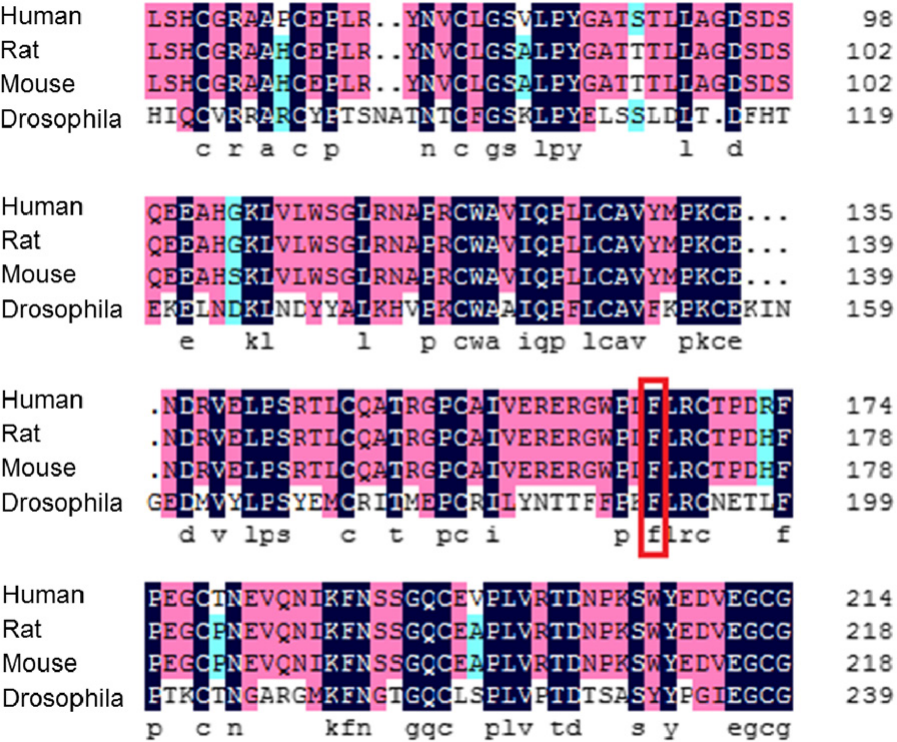
**Conserved amino acids in human, rice, mouse, and*Drosophila*.** Amino acid sequences between human, rice, mouse, and *Drosophila* are similar (t), indicating that amino acid residues are relatively conserved at the mutation site detected (c.T776A).

The first alteration was located at nucleotide position 776 of exon 2, a heterozygous mutation from T to A (Figure 1A); this alteration has not yet been reported by other researchers. We determined that 9 of 12 KCOT patients have this alteration. After comparing the protein amino acid sequences of human, rice, mouse, and *Drosophila* (Figure 2), we concluded that this amino acid is relatively conserved. Meanwhile, following Genomics online software SIFT [37] (http://sift.jcvi.org/) analysis, we concluded that the amino acid change results in a negative and damaging effect on the protein (Provean Preduction: Deleterious; Sift Preduction: Damaging). Through further analysis, we discovered that the amino acid is located in a cysteine-rich domain (CRD) of the Smoothened receptor (Smo) integral membrane protein. The CRD is one of the key players in the HH signaling pathway, and is critical for development, cell growth and migration, as well as stem cell maintenance. The CRD of Smo is conserved in vertebrates and can also be identified in invertebrates. The precise function of the CRD in Smo is unknown. Mutations in the *Drosophila* CRD disrupt Smo activity *in vivo*, while deletion of the CRD in mammalian cells does not appear to affect Smo overexpression [38-40]. Therefore, we believe that this amino acid may tremendously impact development, cell growth and migration, as well as stem cell maintenance. It is important to note that the peak of this alteration is different from mutations at other positions. For example, the peak of A is relatively lower than T, but not as high as half of T. According to a recent report [41], this may be due to the heterogeneity of tumors in issues we detected, indicating that some cells have mutated while others remain unchanged.

The second alteration, a heterozygous mutation from A to G, was located at nucleotide position 862 of exon 3 (Figure 1B) but does not result in an amino acid change at p.G194. The third mutation (c.G1444C, with G/C alleles) was identified in seven tumors (Figure 1C), but does not result in an amino acid change at p.G388. One interesting fact about this synonymous mutation is, with the exception of one sporadic KCOT, all other samples (including sporadic and NBCCS-associated) were identified with this mutation, of which 17 were homozygous and 13 were heterozygous. The frequency of G reached a high of 0.7581, which is far higher than the statistic reported in the single-nucleotide polymorphism (SNP) database curated by the National Centre for Biotechnology Information (NCBI) and the 1000 Genomes Project (minor allele frequency [MAF] = 0.2277). This mutation has been reported in many other cell lines associated with mesothelioma, such as MSTO-211H, NCI-H28, JU77, LO68, NO36, ONE58, and STY51 [42]. Therefore, it is highly likely that this mutation plays a significant role in the occurrence of tumors. However, whether this mutation is related to KCOTs should be determined.

One of the missense mutations (c.G2049C, with G/C alleles), which has been reported previously, has unknown functional implications (Figure 1D). This mutation leads to an amino acid change at p.Ser590Thr; it is important to note that both Val and Gly can be phosphorylated. Meanwhile, SIFT software analysis revealed that an interactive mutation between these amino acids is neutral and tolerated (Table 3). In fact, this altered amino acid is located within the intracellular loops of Smoothened, which may be critically related to downstream proteins, and subsequently impact the functioning of SMO, thereby impacting activation of the HH signaling pathway [43].

**Table 3:** PROVEAN human protein batch result

**Variation**	**Protein sequence change**				**PROVEAN prediction**		**Sift prediction**	
**Row_No.**	**Protein_ID**	**Position**	**Residue_REF**	**Residue_ALT**	**Score**	**Prediction (cutoff = -2.5)**	**Score**	**Prediction (cutoff = 0.05)**
1	NP_005622.1	166	F	I	-5.385	Deleterious	0.002	Damaging
2	NP_005622.1	334	V	G	-5.063	Deleterious	0.003	Damaging
3	NP_005622.1	590	S	T	-1.487	Neutral	0.219	Tolerated

Changes at amino acids 166 and 344 cause a deleterious alteration and a damaging result. The change at amino acid position 590 is relatively neutral and tolerated by the protein.

The missense mutation (c.T1281G, with T/C alleles) has not been reported previously (Figure 1E). This mutation leads to an amino acid change (p.Val334Gly) located on the first extracellular loop of Smoothened, which is closely associated with the function of SMO. Functional studies on transmembrane regions are restricted by technological conditions, but according to the SIFT software (Table 3), mutation of this amino acid leads to damaging changes in the protein; therefore, we believe that this mutation strongly impacts disease occurrence. Additional studies are required to determine whether this mutation causes functional changes in the SMO gene.

The mode of action between the SMO and PTCH genes has yet to be elucidated, but recent studies have shown that any missing gene in yeast will impose pressure on the cell to compensate, thereby leading to additional genetic mutations [44]. Most SMO gene mutations detected are serious alterations, such as missense and synonymous mutations, while frameshift and nonsense mutations have not been detected. Therefore, we believe that the SMO gene mutation may serve as a driving force in patients with KCOTs. To compensate for the defects caused by the SMO mutation, PTCH1 causes the same signaling pathway to mutate, leading to mutation(s) in the PTCH gene. This conjecture needs further work to confirm. In most cases, people believe that tumor growth is driven by the “latest” cancer cell subsets because they carry most cancer mutations. However, many mutations exist at a low frequency, suggesting that tumors contain many subclones the relationships among which are still unclear. A recent report examining the heterogeneity of breast cancer [41] showed that stem cells in breast epithelia could differentiate into luminal and basal cells, which constitute the epithelia. Some also believe that breast neoplasms induced through Wnt1 overexpression are derived from this cell type. Paracrine interaction between two cell types, which is driven by signaling molecules, and its short distance, maintains co-existence of two types of pedigree. Only luminal cells can produce Wnt1, whereas basal cells rely on this protein to proliferate. Wnt1 induces the Hras gene in basal cells of breast neoplasms, which may contain a mutation that drives cancer progression, but this mutation has not yet been detected in cancerous cells in the lumen. There is cooperation between basal and luminal cell clones; this cooperation is necessary for Wnt1 to drive two types of cells in tumors. In addition, KCOTs occur during the early stages of dental epithelial formation, during which dental lamina and its residential, epithelium and stroma interact with others during growth, subsequently inducing differentiation. Therefore, we hypothesize that mutation of some genes in the subcutaneous interstitial tissue may impose pressure on epithelial genes to compensate for the defect, and that the SMO mutation may play a critical role in this process. However, additional studies are needed for confirmation.

As we know, the hedgehog signaling pathway is being suggested to be a drug target for cancer therapy for its activation in human cancers [45-47]. Therefore, we think the two newly identified SMO mutations deserve to be further investigated for their therapeutic application in cancer treatment.

## Conclusions

Following the analysis of SMO gene mutations and combining our results with similar studies, we conclude that SMO plays an important role in the HH signaling pathway and may be responsible for the development of KCOTs and NBCCS. However, further research focusing on the mechanism of their influence on the SHH signaling pathway is required.

## Competing interests

The authors declare that they have no competing interests.

## Authors’ contributions

FC conceived and designed the research. JFQ and YYH provided samples. RZ and LYP performed the experiments, and collected, analyzed and interpreted the data. LYP wrote the manuscript, and edited tables and figures. RZ edited the manuscript, tables and figure legends. FC and TJL reviewed and edited the manuscript. All authors read and approved the final manuscript.
